# The role of the diffusion in the predictions of the classical nucleation theory for quasi-real systems differ in dipole moment value

**DOI:** 10.1038/s41598-022-13715-y

**Published:** 2022-06-10

**Authors:** Kajetan Koperwas, Filip Kaśkosz, Frederic Affouard, Andrzej Grzybowski, Marian Paluch

**Affiliations:** 1 Institute of Physics, University of Silesia in Katowice, 75 Pułku Piechoty 1, 41-500 Chorzów, Poland; 2 Université de Lille, CNRS, INRAE, Centrale Lille, UMR 8207-UMET-Unité Matériaux et Transformations, 59000 Lille, France

**Keywords:** Thermodynamics, Phase transitions and critical phenomena, Structure of solids and liquids

## Abstract

In this paper, we examine the crystallization tendency for two quasi-real systems, which differ exclusively in the dipole moment's value. The main advantage of the studied system is the fact that despite that their structures are entirely identical, they exhibit different physical properties. Hence, the results obtained for one of the proposed model systems cannot be scaled to reproduce the results for another corresponding system, as it can be done for simple model systems, where structural differences are modeled by the different parameters of the intermolecular interactions. Our results show that both examined systems exhibit similar stability behavior below the melting temperature. This finding is contrary to the predictions of the classical nucleation theory, which suggests a significantly higher crystallization tendency for a more polar system. Our studies indicate that the noted discrepancies are caused by the kinetic aspect of the classical nucleation theory, which overestimates the role of diffusion in the nucleation process.

## Introduction

Although the crystallization process is a commonly known phenomenon, the complete understanding of its nature is still far from being achieved. It is mainly due to its complexity, which finally makes that some systems easily crystallize, whereas others do not exhibit any symptoms of crystallization, even at deep undercooling, and finally form the glass. Thus, the complete understanding of this process, including the determination of the physical factors, which govern its occurrence, seems to be a crucial task for contemporary condensed matter physics^1–5^.

Consequently, through the last decades, various theoretical and computational approaches to study the crystallization phenomenon have been proposed. The computational experiments mainly focus on the possibility of the precise calculation of the order parameter, which enables, e.g., the estimation of the time scale, at which the ordered phase within the liquid system appears for the first time (the mean first passage time method)^6–8^. Then, the structure of the formed crystal^9^ and the direct evolution of its size, can be immediately monitored^10^. However, from the experimental point of view, the theoretical methods employing macroscopic features of the system are of more practical importance. Therefore, a variety of theoretical descriptions for the crystallization process have been proposed^11^. Most of them are grounded on the same concept, i.e., the crystal phase starts to spread only if the nuclei of a given (critical) size (and/or shape) are formed^11^. Among those models, the most widely used (probably due to its remarkable simplicity) is the classical nucleation theory (CNT)^5,12,13^. According to the CNT, the critical nuclei's stability is determined by the surface and bulk contributions to the free energy. Consequently, discussed concept predicts that the crystallization process consists of two stages—the formation of the nuclei of critical size (nucleation) and their growth (crystal grow). The first step can be estimated by the number of the nuclei created within the given volume during the fixed time, i.e., the nucleation rate $N$, whereas the second one, i.e., crystal growth rate $U$, describes the velocity of the growth of the crystal structure within the liquid. As a consequence, the overall crystallization can proceed only when $N$ and $U$ are coupled. This simple idea enables explanation of essential experimental observations, i.e., it justifies why some systems can be supercooled up to the glass transitions, whereas others crystallize during cooling, and why some supercooled liquids crystallize during the heating from the glass (it is so called cold crystallization)^14^. In the first case, the separation of $N$ and $U$ plays a key role. The $U$ curve is located closer to the melting temperature than $N$ curve. Hence, at small supercoolings when both components of the crystallization process are substantially separated, $N$ is insufficient to create the stable critical nuclei, which would subsequently growth. On the other hand, at a deeper supercooling, the critical nuclei can be created, but then their growth is suspended by the scarce value of $U$. Finally, the substance does not crystallize. At this point, it must also be noted that a slower cooling rate implies that the system persists at given thermodynamic conditions for a longer time. Therefore, the chance for the creation of (at least one) critical nuclei is higher. Hence, CNT considers the effect of the cooling rate as well. In the second case, when a deeply supercooled liquid is heated, the critical nuclei created at a deep supercooling begin to achieve the temperatures at which $U$ exhibits high values. Thus, we can observe the crystallization process, which previously, during cooling, was unable to take place. Contrary, if $N$ and $U$ curves are close to each other, the optimal temperature range for the crystallization process appears. Then the critical nuclei are formed, and subsequently, they freely grow.

In this paper, on the basis of the two highly similar systems, we challenge the prediction of the CNT. Interestingly, despite the fact that at given supercooling one of studied systems exhibits significantly higher values of the $N$ and $U$, the crystallization event for this system is not observed. Our examinations suggest that the observed inconsistency between CNT predictions and computational experiment results is caused by the differences in the molecular mobility between studied systems. Consequently, we show that the crystallization process's kinetic aspect should not be straightforwardly linked with diffusion, as CNT assumes.

The CNT has frequently been using for the theoretical description of experimental and computational experiments through the last decades. However, the computational experiments deserve particular attention because it enables to examine the crystallization tendencies on the most fundamental level of intermolecular interactions. For this purpose, the simple model systems characterized by the well-defined intermolecular potentials can be used. The most frequently studied systems are those in which pairwise intermolecular interactions are described by the Lennard–Jones potential or its approximation valid at short distances, i.e., the soft-sphere potential^15–18^. The mentioned choice is justified by the fact that the Lennard–Jones potential can be theoretically derived on the basis of the interactions between permanent and induced dipole moments. Consequently, those simple model systems were used to verification of the CNT^19–21^ as well as also to study the influence of the attractive and repulsive intermolecular interactions on the crystallization tendency^22–28^. Reported studies suggest that the increase in the repulsion results in the decrease in the nucleation barrier and interfacial free energy^29^. The other examinations focused on the role of the attraction in the crystallization process, deliver the conclusion on the positive impact of the intermolecular attraction on the reduction of the time needed for the crystallization at given temperature^30^. Simultaneously the different approach, i.e., the computational studies performed on the hard molecules, revealed that the molecular anisotropy ignored by simple model systems is crucial in determining the phase diagram of the system^31–36^. However, it must be mentioned that for hard molecules, the temperature enters the thermodynamics only in a trivial way^37^. Consequently, the alternative models, which consider the interactions between non-spherical molecules have been developed, e.g., Kihara^38^ potential, the Gaussian overlap model^39^, and the Gay-Berne potential^40^, and prove the important role of the structural anisotropy in the thermodynamics and dynamics of the studied systems. However, from the experimental point of view, the most natural is the all atom–atom (or site–site) interactions approach, which unfortunately requires much more computational effort^41^. Nevertheless, the all atom–atom approach makes that the structure of complex molecules can be reflected, and therefore, the closing agreement with the experiments may be expected. As a consequence, the structural^41–44^ and dynamical^45–49^ properties of many model system have been deeply examined concluding that this approach can be successfully applied for slightly non-spherical molecules^50^. Following this result, the very recent study reports that the permanent dipole moment orientation within the anisotropic molecules is of crucial importance for the crystallization process. The two analogical systems, which vary exclusively in the orientations of the dipole moment, exhibit entirely different stability behavior despite that the same isobaric conditions and identical cooling rates are applied^51^. Briefly speaking, the perpendicular to the longest molecular axis orientation of the dipole moment favors crystallization. In contrast, the deep supercooling of the system with parallel to the longest molecular axis orientation of the dipole moment is easily achieved. This outcome is not only relevant from the experimental point of view, but it is also important for further computational studies because it emphasizes the practical utility of the model systems tested therein. Only slight modifications of the molecular architecture result in drastically different crystallization tendency. Hence, model systems from Ref.^51^, which comprise the so-called quasi-real molecules, seem to be promising candidates to examine the crystallization process and then the predictions of the CNT. At this point, it is also worth justifying that the use of the quasi-real molecules, i.e., the molecules which mimic the real ones but cannot exist in reality, helps to eliminate the uncontrolled effects of various intramolecular factors on the considered process or physical quantity. At this point it is also worth mentioning that in contrast to simple models, the results obtained for one of the quasi-real systems cannot be appropriately scaled to reproduce results registered for another system. Thus study on quasi-real systems provides promising alternative to typical computational experiments.

## Results

**Figure 1:**
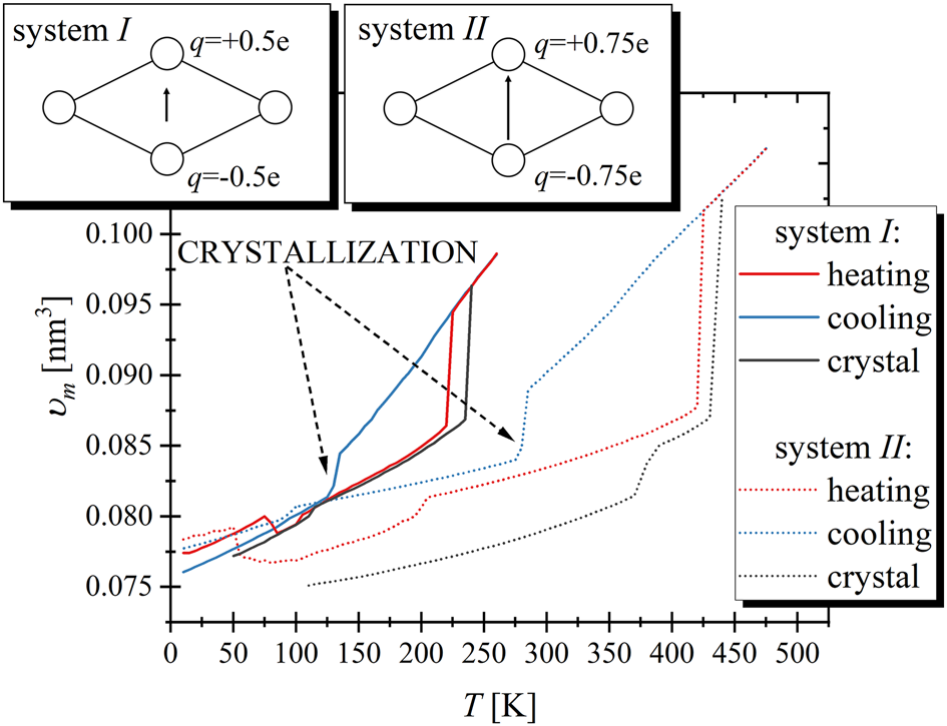
The temperature dependences of the volume for two studied model systems registered during heating and cooling are shown. The black lines represent the heating of the determined crystal structure. In the insets, the schemes of the structures of RM are presented.

Similarly, to our previous examinations of the system *I*, we began the studies of system *II* from the constructing the perfect FCC crystal structure constructed from 2048 molecules and heating it from $10$ K up to the temperature which is about $50$ K higher than the temperature at which we observe a rapid increase in the volume, see Fig. 1.

On the basis of our recent results for the system *I*, we can state that the rapid increase in the volume from about $0.086$ to $0.094{\mathrm{ nm}}^{3}$, which is observed around $T=220 \mathrm{K}$, indicates on the melting of the crystal structure. Therefore, the thermodynamic conditions at which the system *II* is in the liquid phase can be recognized in the similar way, see increase in volume from about $0.088$ to $0.101{\mathrm{ nm}}^{3}$ at $T=420 \mathrm{K}.$ Next, we cooled the systems up to the starting temperatures. During this process the drop in the volume can be observed. The latter indicates on occurrence of the crystallization process, which takes place at ${T}_{cr}=130$ and $280\mathrm{ K}$, respectively for the systems I and *II*.

According to CNT the nucleation rate is expressed as follows^13^

1 $$N= {\rho }_{liq}^{4/3}\sqrt{\frac{\gamma }{{k}_{B}T}}Dexp\left(-\frac{\Delta W}{{k}_{B}T}\right),$$

where ${\rho }_{liq}$ is the number density of the liquid, $D$ is a diffusion constant, ${k}_{B}$ is the Boltzmann constant, and $\Delta W=\frac{16}{3}\frac{{\gamma }^{3}}{{\left(\Delta {G}_{\upsilon }\right)}^{2}}$ is the nucleation barrier, in which $\Delta {G}_{\upsilon }$ denotes the driving force per volume unit (i.e., the difference between Gibbs free energy for liquid and bulk phases) and $\gamma$ is the interfacial free energy (IFE). The next physical quantity determining the occurrence of the overall crystallization process is the crystal growth rate, role of which can be computed by the following expression

2 $$U={A}_{U}\left(T\right)\cdot f\left(T\right)\cdot \left[1-exp\left(\frac{-\Delta G}{{k}_{B}T}\right)\right],$$

where ${A}_{U}\left(T\right)$ describes the molecular mobility and can be approximated by $D\cdot a/{\lambda }^{2}$, in which $a$ is the average width of the crystal lattice spacing ($a\approx {\rho }_{cr}^\frac{1}{3}$, ${\rho }_{cr}$ denotes the number density of the crystal), $\lambda \approx {\rho }_{liq}^{-1/3}$ is the atomic jump distance, whilst $f\left(T\right)$ describes the grow mechanism, which for the normal growth $\approx 1.$ For all thermodynamic conditions at which during the cooling systems remain in the liquid phase the diffusion constant is determined using the GROMACS software on the basis of the mean square displacement calculated for atoms for long times. In this way both translational and rotational contributions to the molecular motion are considered. Subsequently, the estimated dependences $D\left(T\right)$ are approximated by the Vogel–Fulcher–Tammann equation for the needs of further analysis (see Fig. 5a, which we discussed later). To estimate the value of $\Delta G$ we use the method proposed by Gutzow^52^ with the redefined integration pathways^53^, according to which, at isobaric conditions, the driving force for the crystallization takes the following form $\Delta G\left(T\right)=-{\int }_{{T}_{m}}^{T}\Delta S\left(T\right)dT$, where $\Delta S$ is the difference in the entropy between the liquid and the solid phases. Taking into account the melting boundary conditions $\Delta {S}_{m}=\Delta {H}_{m}/{T}_{m}$, $\Delta S$ can be calculated using obtained directly from simulation-runs values of the enthalpy $H\left(T\right)$ and the classical relation between enthalpy and the entropy, $T={\left(\partial H/\partial S\right)}_{p}$. The estimation of the melting temperature has been done using the liquid–solid coexistence method. In this order, we visualized the structure up to which each system crystallizes. Then, we determined the fragments characterized by a high degree of order, which for both systems are characterized by the triclinic shape and consist of molecules placed in corners. Based on the latter, we constructed another crystal structure and equilibrate it at the temperature close to ${T}_{cr}$ for both systems. Since we observed that the small defects occur again, we selected the set of 5 × 5 × 5 molecules within which the created crystal structures were highly ordered and those crystal fragments are used for further examination. On their basis, we construct the crystal structures consisted of 2250 molecules, and equilibrate it at the temperatures significantly smaller than ${T}_{cr}$. Subsequently, we heat the systems to confirm that the crystals are stable at higher temperatures. The results are presented in Fig. 1 of the main text. It can be seen that created crystal structures do not tend to melt, although, during the heating process, the tiny and step increase in volume is detected for both systems. Probably, these changes of volume are results of the transformation to the different polymorphic form, which is more stable at higher temperatures. This scenario seems to be supported by the evident visible change in the temperature dependence of volume, which is observed around $100\mathrm{ K}$ during the cooling of the system *II*. However, to confirm this suspicion further researches are required. Nevertheless, it is worth mentioning that created structures are more stable at higher temperatures than the ones resulted from starting FCC configuration. This fact encourages that the crystal structures established by us can be used to determine ${T}_{m}$. Hence, we constructed the special biphasic simulation box, within which $3456$ molecules had been equally divided between the crystal and liquid phases separated by a small gap. Due to the fact that at melting conditions, the solid and liquid phases remain in the thermodynamic equilibrium, ${T}_{m}$ can be determined by the examination of the behavior of the biphasic system. The performed simulations of biphasic box last for $5ns$ and have been done at range of temperatures differ by $1\mathrm{ K}$. Following the visual examination of the obtained configurations we determine that melting temperatures, which equal ${T}_{m}=150 \mathrm{K}$ and ${T}_{m}=326 \mathrm{K}$ for the systems *I* and *II*, respectively. At this point, we have to comment that in our previous studies^51^ we determined the ${T}_{m}$ for the system *I*, using liquid–solid coexistence method as well, and we obtained that ${T}_{m}=194 \mathrm{K}$. However, in that experiment we did not determine the crystal structure. Instead of that, we employed the structure up to which the liquid had crystalized. Consequently, we probably employed the structure stable at higher temperatures instead of the one, which is the most energetically optimal. However, in this work, we intend to focus on the most fundamental case, i.e., we estimate the crystallization tendency against the desired (and the most energetically optimal) structure. In this context, it is worth noting that the values of ${T}_{m}$ estimated herein are in similar relations to those of ${T}_{cr}$ and also to the temperature at which initial crystals melt, which suggests that the determined structures are mutually appropriate.

**Figure 2:**
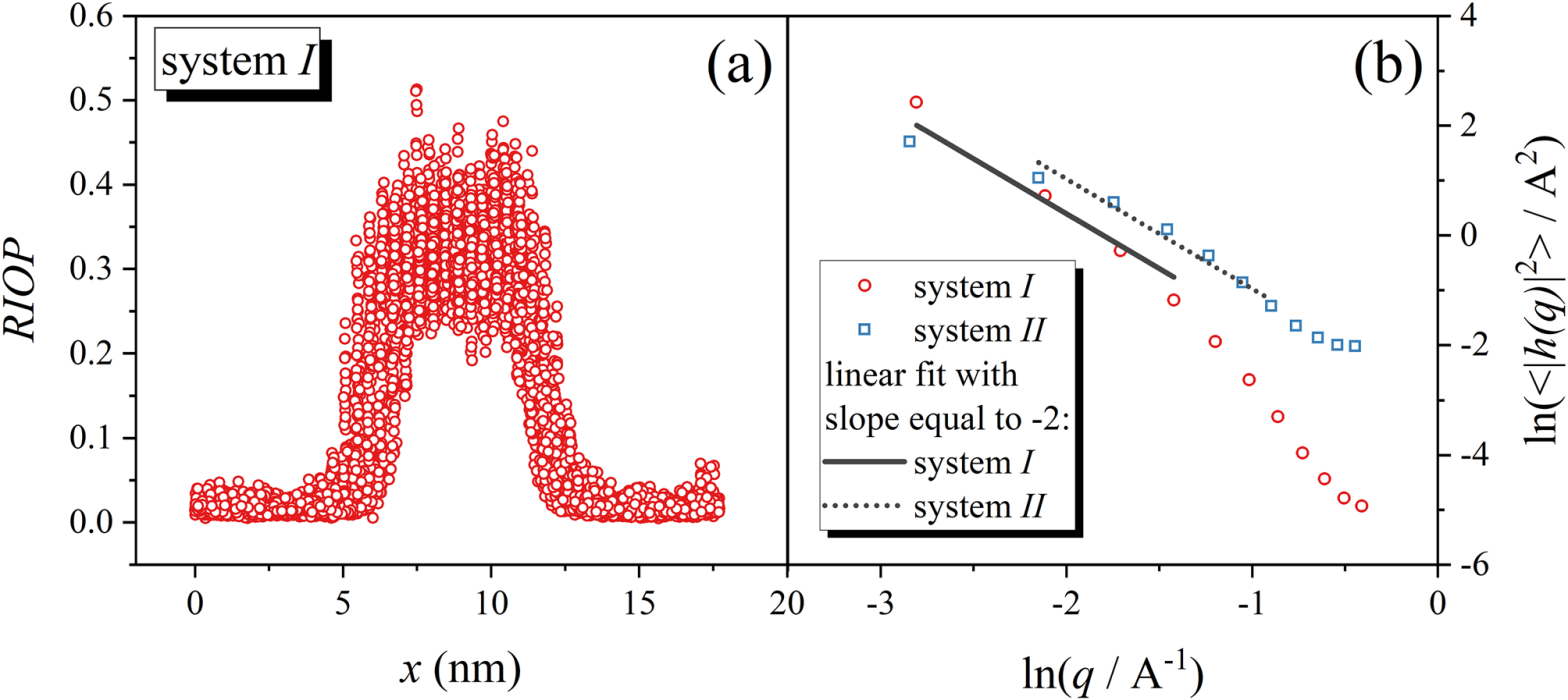
In the panel (**a**), the value of the rotational-invariant order parameter along the direction perpendicular to the solid–liquid interface for the system *I* is presented. In the panel (**b**), the fluctuation spectrum of the interface height for two RLM systems is shown. The straight lines represent the fit of the linear function with a constant slope equal to − 2.

However, the most challenging is the determination of the $\gamma$. Fortunately, the special computational method for calculation of $\gamma$ have been proposed. The two main approaches are the cleaving potential method^54–56^ and the capillary fluctuation method^57–61^. Despite that both methods are applicable only at the melting conditions, they strongly differ in the way of work. In the cleaving potential method, the biphasic solid–liquid system is transformed into two separate systems (liquid and solid) by means of external potentials. Then, $\gamma$ is estimated on the basis of the work which is performed by those potentials during the transformation process. However, the precise application of this method is associated with some technical difficulties. It is because the reversibility of the transformation process must be ensured, and therefore, the accurate control on the transformation process is needed^62^. Alternatively, $\gamma$ can be calculated in the more direct way using the capillary fluctuation method (CFM), which requires only one simulation run, during which any knowledge of the complex process of the interface creation from separated bulk systems is not needed. Instead of that, through the simulation run, the fluctuations of the interface are measured. It enables the estimation of the interface stiffness, which is related to $\gamma$. The remarkable advantage of CFM is the fact that it considers the anisotropy of the interface, whereas the cleaving method is recognized as more accurate. Till now, both methods have been applied to calculate $\gamma$ values for model systems such as hard-spheres^55,63^ and Lennard–Jones^25,56,60^. It must be however noted that for the real materials the CFM is more often employed, which is mainly stimulated by the ease of its application. Consequently, using the CFM the $\gamma$ values have been calculated for metallic compounds^57–59,64^, alloys^65,66^, and a few molecular systems^67^ including pharmaceuticals^68–70^. Hence we decided to employ CFM to determine $\gamma$ for studied herein systems. The use of CFM requires creation of the biphasic box. However, the considered solid–liquid interface must be the quasi-one-dimensional, and therefore the special geometrical conditions of the simulation box have to be ensured, i.e., when interface is perpendicular to the length of the system, ${L}_{x}$, its thickness must be much smaller than its width, ${L}_{z}\ll {L}_{y}$. Then the interface fluctuates only in the one dimension ($x$). Consequently, we construct the box containing $5000$ RLM divided equally to the crystal and liquid phases. It is also worth mentioning that due to boundary conditions the simulation of the biphasic system implies the existence of two interfaces, of which the fluctuations magnitudes are studied. The convenient way used to determine the temporary position of the interface is the calculation of the rotational-invariant order parameter^64,71–75^ ($RIOP$) for geometrical center of the molecules. The $RIOP$ enables the distinction between solid-like and liquid-like molecules, because the solid-like molecules are characterized by the significantly higher values of the order parameter. The example of obtained results is presented in Fig. 2a, where the calculated $RIOP$ for each molecule of system *I* is plotted as a function of the position of the molecule in the dimension perpendicular to the interface plane ($x$ direction).

As we already mentioned the liquid-like and the solid like molecules can be clearly distinct. The evolution of the RIOP can be described by the following function $RIOP\left(y\right)=\frac{{o}_{s}+{o}_{l}}{2}+\frac{{o}_{s}-{o}_{l}}{2}\left[tanh\left(\frac{y-{h}_{1}\left(x\right)}{{\delta }_{1}}\right)+tanh\left(\frac{y-{h}_{2}\left(x\right)}{{\delta }_{2}}\right)\right]$, where ${o}_{s,l}$ are the average values of the RIOP in the solid and liquid, ${\delta }_{\mathrm{1,2}}$ are effective widths of the interfaces, and ${h}_{\mathrm{1,2}}\left(x\right)$ are functions describing the positions of the interfaces in capillaries, i.e., sections from $x$ to $x+\Delta x$, which are orthogonal to the interface. During the simulation run the $h\left(x\right)$ describes the interface fluctuations. The latter can be Fourier-transformed leading to the following expression for power spectrum of the quasi-one dimensional interface $\langle {\left|h\left(q\right)\right|}^{2}\rangle =\frac{{k}_{B}{T}_{m}}{{L}_{x}{L}_{z}\stackrel{\sim }{{\gamma }_{m}}{q}^{2}}$, where $h\left(q\right)$ is the one-dimensional Fourier transform of $h\left(x\right)$ with $q$ as the wave number, $\langle \rangle$ denotes the time average, ${k}_{B}$ is the Boltzman constant, and ${L}_{x}$,${L}_{z}$ are width and thickness of the simulation box. The interfacial stiffness, $\stackrel{\sim }{{\gamma }_{m}}$, is used as a fair estimation of the ${\gamma }_{m}$, whereas different orientations of the crystal structure enable the determination of ${\gamma }_{m}$ anisotropy. Nevertheless, at this point, it is worth mentioning that the direct studies of model^57,59,60,76^ and realistic^62,67,77,78^ systems suggest that this effect is usually relatively weak, and therefore ${\gamma }_{m}$ can be obtained from $\stackrel{\sim }{{\gamma }_{m}}$ determined from a single crystallographic orientation. Then, $ln\left(\langle {\left|h\left(q\right)\right|}^{2}\rangle \right)$ is a linear function of $ln\left(q\right)$ with a slope equal to − 2^57^, and the intercept is directly related to $\stackrel{\sim }{{\gamma }_{m}}$. Thus, ${\gamma }_{m}$ can be estimated by fitting the obtained dependence of $\langle {\left|h\left(q\right)\right|}^{2}\rangle$ on $q$ (expressed in logarithmic scales) to the linear function with the constant slope equal to − 2 and analyzing its intercept, see Fig. 2b, where discussed fits are presented for both studied systems. As the initial stage of this analysis we determined the region at which $\langle {\left|h\left(q\right)\right|}^{2}\rangle$ is characterized by the linear dependence on $ln\left(q\right)$ with slope equal to − 2. Subsequently the linear function was adjusted to the chosen points. Estimated in this way values of ${\gamma }_{m}$ are equal to $2.53\pm 0.44\mathrm{ mJ}/{\mathrm{m}}^{2}$ and $4.15\pm 0.29\mathrm{ mJ}/{\mathrm{m}}^{2}$ respectively for the systems *I* and *II*. Subsequently, the temperature dependence of $\gamma$ was estimated according the Turnbull law^79^,

$$\gamma \left(T\right)={\gamma }_{m}{\left(\frac{{\rho }_{cr}\left(T\right)}{{\rho }_{cr}\left({T}_{m}\right)}\right)}^{2/3}\left(\frac{\Delta H\left(T\right)}{\Delta {H}_{m}}\right).$$

**Figure 3:**
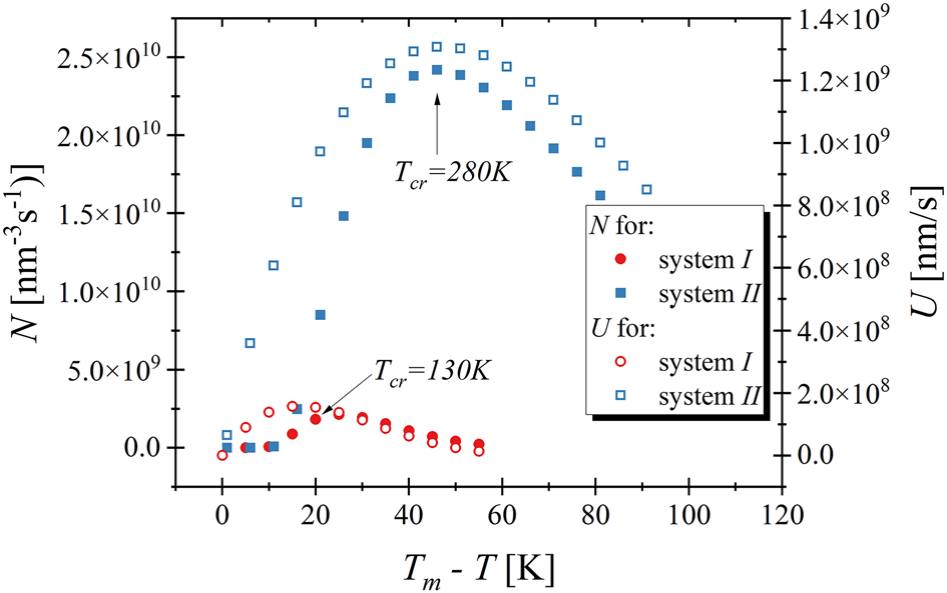
The values of the nucleation and crystal growth rates for different temperatures are shown. The arrows indicate *N* and *U* value obtained at the temperature at which the studied RM system crystallized during cooling.

Finally, the calculated values of the $N$ and $U$ are presented in Fig. 3.

It can be clearly seen that $N$ and $U$ possess maxima located close to each other for both studied systems. Interestingly, the above maxima are located around the temperature at which the given system crystallizes during cooling. Arrows in Fig. 3 indicate the mentioned temperatures. Thus, CNT fairly predicts the thermodynamic conditions of crystallization. However, at this point, it is worth noting that despite the fact that both systems are very similar and that the procedures of the performed experiments are identical the system *II* crystallizes at temperatures much lower than ${T}_{m}$ (${T}_{m}-T=45 \mathrm{K}$ for system *I*, whereas for system *II* this difference equals $20\mathrm{ K}$). This observation is even more intriguing when one takes into account that around $20 \mathrm{K}$ below the melting temperature, $N$ for system *II* is about 3 times higher than for system *I*. Additionally, at discussed thermodynamic conditions $U$ for system *II* is also much higher than for system *I,* and therefore from the CNT point of view, neither $N$ nor $U$ suspend the crystallization. Consequently, the system *II* should easily crystallize much faster than it is observed during cooling experiments. Moreover, we would like to put the reader’s attention on another intriguing fact. Examining the cooling procedure, one might observe that despite the fact that at ${T}_{cr}$ the system *II* possesses almost 10 times higher maximal values of the $N$ and $U$, the time needed to crystallization of both systems are similar, i.e., the initial liquid structures become entirely solid within the same simulation time ($10$ ns).

## Discussion

**Figure 4:**
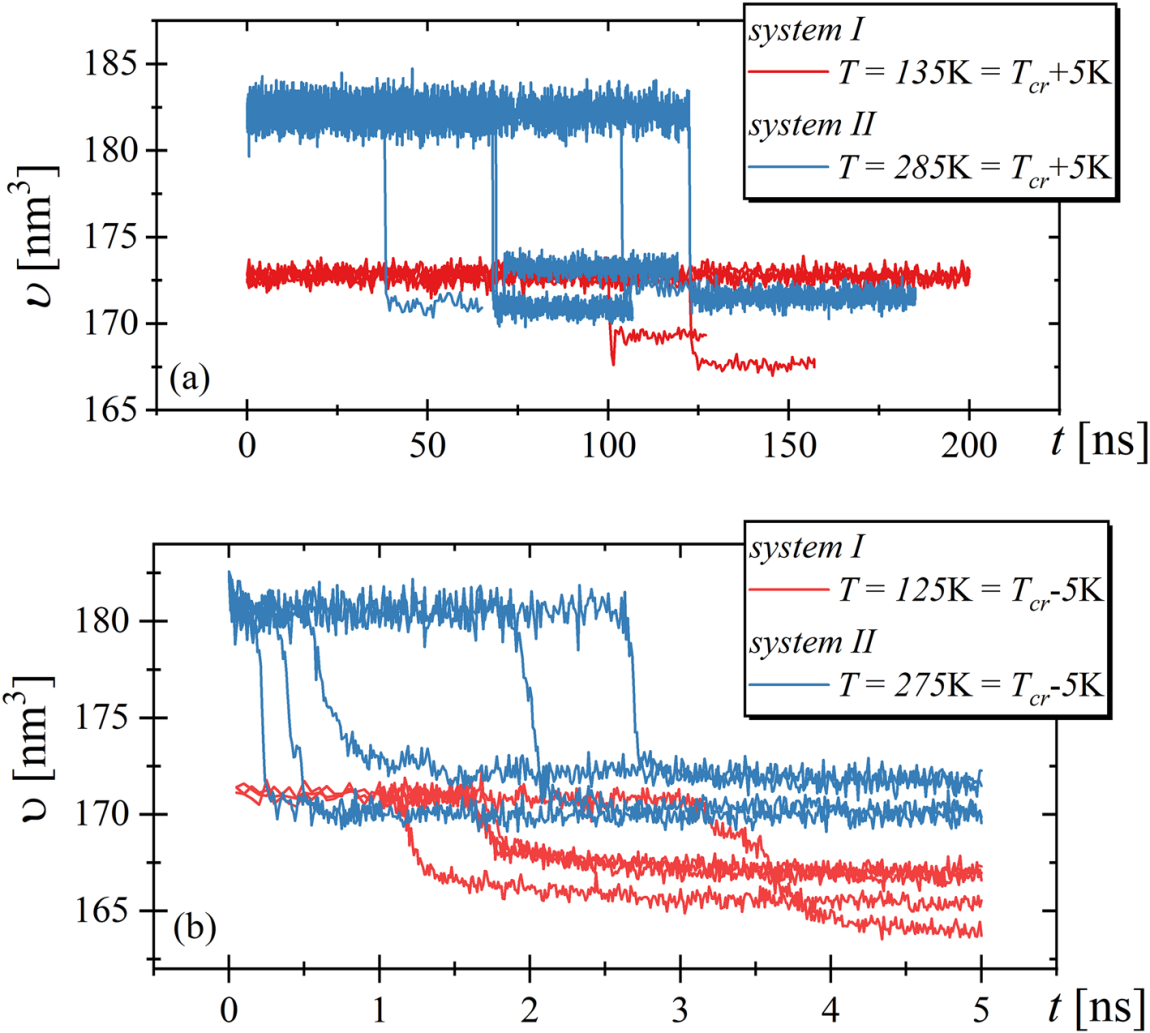
The time evolution of systems volumes at temperatures $5$ K higher (top) and lower (bottom) than the temperature at which RMs systems crystallized during cooling.

Nevertheless, it must be noted that the crystallization process starts from the stochastic formation of the critical nuclei within the liquid. Therefore, to examine the crystallization tendency in detail, and then to confirm that the characteristics of crystallization process for examining systems are indeed similar we simulate the liquid structure at temperature $5 \mathrm{K}$ higher than ${T}_{cr}$ for $200 \mathrm{ns}$ or till the time at which we observed the crystallization event. The results are presented in Fig. 4a, where one can see that increase in the temperature implies an extension of the time needed for registration of the crystallization for both systems.

**Figure 5:**
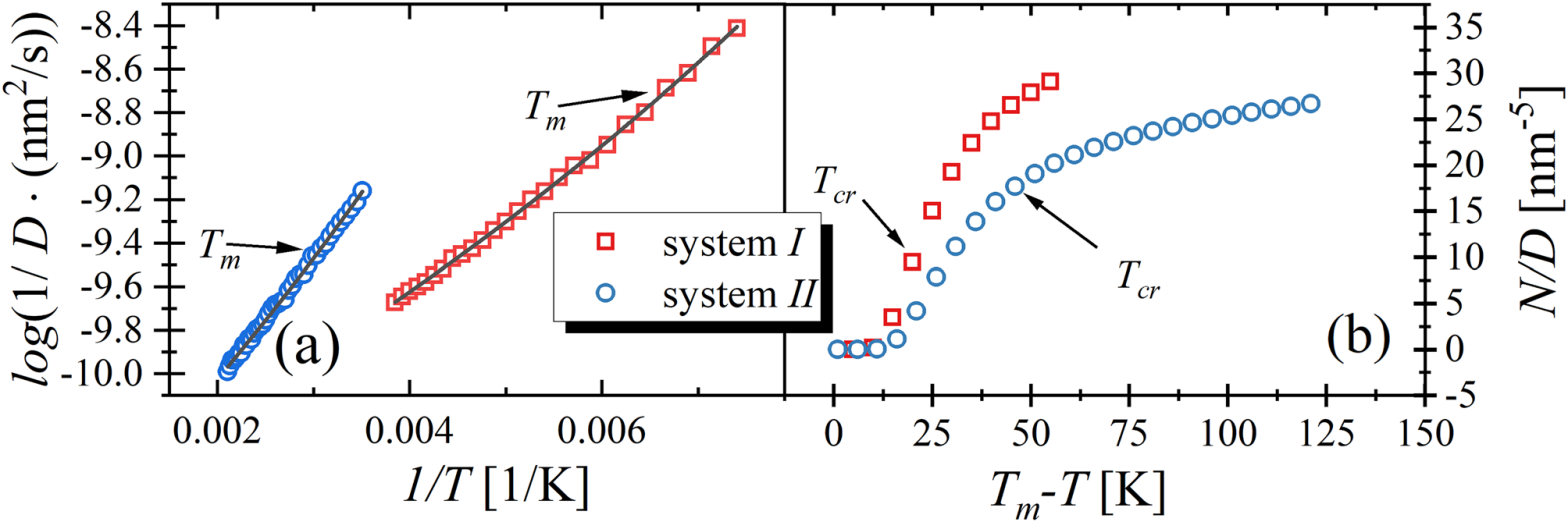
In the left panel the temperature dependences of diffusion constant for RMs systems are presented. The black lines represent the fit of the VFT equation, $\mathrm{log}\left(\frac{1}{D}\right)=\mathrm{log}\left(\frac{1}{{D}_{0}}\right)+log\left(e\right)(\frac{B}{T-{T}_{0}})$. The fit parameters are $\mathrm{log}\left(\frac{1}{{D}_{0}}\right)=-(1068\pm 4){10}^{-2}$, $B=\left(52\pm 3\right){10}^{1}\mathrm{ K}$, ${\mathrm{T}}_{0}=35\pm 4\mathrm{ K}$ for system *I* and $\mathrm{log}\left(\frac{1}{{D}_{0}}\right)=-(1110\pm 7){10}^{-2}$, $B=\left(12\pm 1\right){10}^{2} \mathrm{K}$, ${T}_{0}=17\pm 2\mathrm{ K}$ for system *II.* The right panel shows the values of the nucleation rate divided by the diffusion constant for studied systems.

This observation corresponds with a prediction of the CNT. Additionally, it is worth mentioning that system *I* is more sensitive for applied temperature changes because 3 from 5 simulation runs did not end in the crystallization, whereas system *II* always crystallized. Summarizing, we can state that at temperature equals ${T}_{cr}+5K$ the time needed for the registration of crystallization is slightly shorter for system *II.* However, at discussed temperature conditions the nucleation rate is more than 25 times higher for system *II* than for system *I*. It implies that assuming that the time needed for crystallization is equal to about $100ns$ for system *II* (see Fig. 4a), we could anticipate that system *I* would persist in liquid state for $2500 \mathrm{ns}$ (the total time would be 25 time longer than 100 ns). However, within only $200 \mathrm{ns}$, the system *I* crystallized twice. Thus, comparing both systems the prediction of CNT does not correspond to the observed results. Subsequently, we simulated both systems at temperature $5 \mathrm{K}$ lower than ${T}_{cr}$. The results are shown in Fig. 4b. As one can observe both systems always crystallize within 5 ns, i.e., within the time which is 2 times shorter than in the case of cooling experiment. Similar to previous results the crystallization process proceeds slightly faster for system *II*. However, in this case, the differences between both systems are less prominent. Hence, at temperatures $5 \mathrm{K}$ lower than ${T}_{cr}$ the stability behaviors of studied systems can be considered as comparable. Moreover, we would like to note that CNT predicts that at temperature lower than ${T}_{cr}$ the crystallization process for system *II* should slow down due to the decrease in $N$ and $U$, which is not observed in the performed experiments, see Fig. 5b.

At this point it has to be noted that $N$ values presented in Fig. 3 are expressed in the unit of $1/{\mathrm{nm}}^{3}\mathrm{ s}$, which implies that $N$, and hence the number of the created critical nucleuses depends on the system size. The two studied systems, which are comprised of the same amount of the molecules, are simulated at various temperatures. Therefore, they exhibit different volumes. Nevertheless, as it can be seen in Fig. 4, the differences in $\upsilon$ equal only about 5% and therefore its impact on $N$ can be neglected.

Putting an attention on Eqs. (1) and (2), the possible explanations of observed differences between prediction of CNT and computational experiment can be found. Both equations consider the diffusion of the molecules. It immediately implies that systems possessing a higher value of $D$ exhibit higher values of $N$ and $U$. It is crucial in the case of comparison between systems which are characterized by the significant differences in ${T}_{m}$ because the diffusion strongly depends on the temperature. The higher ${T}_{m}$ implies the faster diffusion of the system and consequently the greater $N$ and $U$ values are predicted by CNT. The latter seems to be crucial especially in the case of the system characterized by similar structure. In Fig. 5a one can see that $D\left({T}_{m}\right)$ for system *II* is higher for about one decade than for system *I*.

The latter immediately implies 10 times higher values of $N$ and $U$ for system *II*. Interestingly, one can see in Fig. 3 that $N$ and $U$ between both systems differ also about 10 times. Hence, the reported variation in CNT predictions for both systems is consistent with differences in $D$. Following this observation, in Fig. 5b we present $N/D$ values for both systems, which indeed are very similar. This finding confirms that the main reason for divergences in CNT predictions for studied herein system is the noticeable difference in $D$ values. At this point it is worth mentioning that for liquid close to the melting conditions the ratio between rotational and translational diffusion is constant and independent on the temperature^80^. Hence, neither translation nor rotation can be treated as a limiting factor for crystallization process of system *I*.

Summarizing, in this paper we calculate the $N$ and $U$ curves according to CNT for two RM systems, which differ exclusively in the value of the dipole moment. The use of proposed model molecules enables entire elimination of the molecular structure role in the crystallization process. Importantly, it makes also that, in contrast to standard simple model systems, obtained results for the system *I* cannot be uses to reproduce the results determined for the system *II*. It is also worth noting that, we calculate $\gamma$ using CFM, instead of estimation of its value. Our results show that $N$ and $U$ curves differ strongly for two studied system. The system with higher value of the dipole moment is characterized by about 10 times higher $N$ and $U$. Interestingly, despite the fact that the system *II* exhibits drastically higher values of $N$ and $U$, it does not crystallize at expected thermodynamic conditions i.e., at conditions at which $N$ and $U$ for second system are sufficient to observe the crystallization process. Our results suggest that the main reason for observed discrepancies between results of performed computational experiments and CNT predictions is the diffusion constant.

## Methods

We employ the previously proposed the quasi-real molecules of the rhombus shape, i.e., rhombus-like molecules (RMs), which remarkable advantage is that keeping the simplicity of classical model systems, they display the structural anisotropy typical for the real molecules and simultaneously enable the creation of the differently oriented dipole moments, $\mu$^51,81–83^. On the basis of the results reported in Ref.^51^, we know that only one of the 5 different systems, which vary in the values and orientation of $\mu$, crystallizes. Therefore, we use this system as a reference one. It consists of 4 identical atoms (of carbon atom mass) arranged, as we already mentioned, in a rhombus shape, which implies that RM possess short and long molecular axes (along diagonals of rhombus) simultaneously keeping identical bonds lengths. The latter is set to equal 0.14982 nm, which is close to 0.14 nm, i.e., a length of the bond linking two carbon atoms in the benzene ring. Additionally, the angles between bonds in RM are established to make one diagonal two times longer than the other. To ensure the best mimic of the real molecules by RM, the stiffness of bonds, angles, and dihedrals, as well as the non-bonded interaction between atoms of different RM molecules, are defined by OPLS all-atom force field parameters^84^ provided for carbon atoms of the benzene ring. Then, the permanent $\mu$ is obtained by redefining charges of given atoms, i.e., those arranged along the longer axis are set to $0.0e$ ($e$ is an elementary charge), whereas those places along shorter one equal $\pm 0.5e$. In this way, we obtain the reference system *I* (i.e., system *C2* from Ref.^51^). The second examined herein system, i.e., system *II*, is identical to the previous one except of the difference in charges’ values, which are set to $\pm 0.75e$ for system *II*. Consequently, the two model systems differ only in the value of the dipole moment, ${\mu }_{II}=\frac{3}{2}{\mu }_{I}$. Schemes of two studied molecules are presented in the insets of Fig. 1. At this point is worth recalling that consistently to our previous mention the applied method for RM molecules creation makes results obtained for one of them cannot be somehow used to reproduce the results for another one. However, the identity of the molecular structure is preserved.

The heating process was performed by the use of the GROMACS software^85–88^ at conditions of constant temperature and pressure, which were controlled by the Nose–Hoover thermostat^89–91^ and Martyna–Tuckerman–Tobias–Klein barostat^92,93^ ($p=1000 \mathrm{bar}$) respectively. The increase in the temperature between subsequent steps is equal to $5 \mathrm{K}$. The first half of simulations, which last for $5$ ns (the time step $dt=0.001$ ps), was spared for equilibration of the system, whilst the data was collected during the second half.
